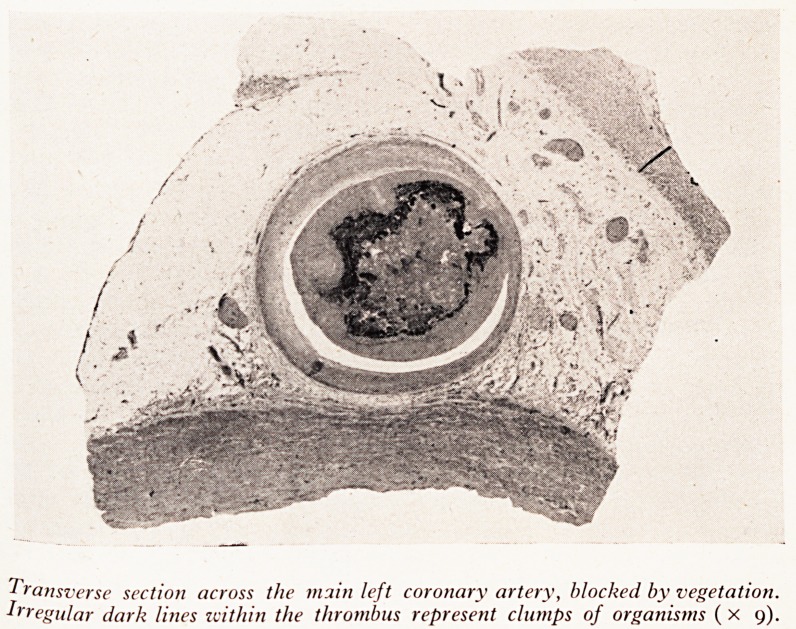# Clonorchiasis

**Published:** 1961-04

**Authors:** T. F. Hewer


### CLONORCHIASIS

*Clinico-Pathological Conference held on 22nd November, 1960*

CHAIRMAN: PROFESSOR T. F. HEWER

(*P.M. No.* 6636)

*Mr. Peacock*: This patient was a 56-year old Chinaman, a seaman, and a native of Hong Kong, who was transferred as an emergency case by air to us on the 14th July. The provisional diagnosis was that this was a case of portal hypertension with uncontrolled bleeding from oesophageal varices. The history of this particular attack commenced in May, 1960, when he was admitted as a case of haematemesis to the Royal Victoria Hospital, Boscombe. At that time he had a Hb of 33 per cent but he was a known case, even at that time, of portal hypertension with oesophageal varices, and he was also incidentally known to have a chronic gastric ulcer. The investigations indicated that his liver function tests were abnormal, the serum albumin being 2·9 gm per cent, globulin 3 gm per cent, and the albumin: globulin ratio 1:1. His serum bilirubin was 2·5 mgm per cent and his alkaline phosphatase 11 units. Examination also revealed the presence of ova of *Clonorchis sinensis*. He was treated at that time by a blood transfusion and was transferred to the Royal Free Hospital on 30th June under the care of Professor Sherlock. While he was under investigation at the Royal Free Hospital he had two further haematemeses which were controlled by the use of a Sengstacken tube, intravenous pitressin and blood transfusion.

His previous history, apart from this incident, was that he was known to have suffered from beri-beri in 1942 and his first haematemesis had been in Hong Kong in 1953. He had a further haematemesis in 1955 which was treated in Hong Kong by splenectomy. In September, 1959, whilst in London, he had three further haematemeses and was investigated at the Hospital for Tropical Diseases, where it was shown that he had advanced cirrhosis of the liver, with large oesophageal varices and also that he had a large gastric ulcer and *Clonorchis sinensis* ova in his stools.

When he arrived at the B.R.I. his general condition was satisfactory and his B.P. normal. He had a Sengstacken tube into his stomach and oesophagus and his bleeding was controlled. The following morning we operated on this patient, through a right thoraco-abdominal incision, removing the eighth rib, and performed a preliminary portal venogram which indicated that he had a patent portal vein. His portal pressure at that time was 300 mm. water, which is about 120 mm. above normal. The portal vein was exposed and transected and at the point of transection it was found that there were three lumina. This seemed to me to indicate that this was a case in which there had been previous venous thrombosis which had recanalized into three separate channels. I excised the three channels and converted them into one main channel and did an end-to-side portocaval anastomosis. The liver was markedly cirrhotic with numerous regenerating nodules, the gall bladder contained thick turbid bile with a single gall stone, and there was a large chronic gastric ulcer on the lesser curvature of the stomach which clinically appeared possibly, although not definitely, malignant. At the closure of the operation a liver biopsy was taken.

*Dr. Sanerkin:* I examined the biopsy at the B.R.I. and considered it was fairly typical of portal cirrhosis except for two very unusual features. There was an intense eosinophilic infiltration which suggested some sort of parasitic infestation. There was also a largish bile duct with marked mucous-gland proliferation around it, which is said to occur typically in clonorchiasis; but of course I could not make a definite diagnosis of that.

*Mr. Peacock:* For the first 36 hours after the operation the patient's condition was satisfactory, but after that time he developed a temperature of 104° F with rigors, and a blood culture taken at that time yielded a profuse growth of *Staph. aureus* which was coagulase positive.

Before the operation was performed he had been placed on penicillin and streptomycin cover. When we found he had a positive blood culture he was immediately given in addition intravenous erythromycin and the infection appeared to be partially controlled. At the same time we noticed that there was a purulent discharge from the puncture in his arm where he had a polythene cannula inserted at the time he was transferred from the Royal Free Hospital The tip of the catheter lay in the axillary vein and he had been transfused through that. The catheter was immediately removed and subsequently culture from the puncture hole also revealed a coagulase positive *Staphylococcus aureus.* During the next 48 hours his general condition deteriorated. He developed fairly severe hypotension which was only partially controlled by the use of hydrocortisone and noradrenaline. On the 20th July at 5 o'clock he suddenly became dyspnoeic with pain in the chest, his B.P. fell to 70 mm Hg and he died in a short time. We thought at that time that the cause of death was either pulmonary embolus or a coronary thrombosis.

*Professor Hewer:* Is there any difficulty about the use of cortisone in the presence of a more serious staphylococcal infection? Did you feel that was a problem?

*Mr. Peacock:* I didn't. As a matter of fact in the post-operative period I was not there; I was on holiday. I really do not know whether I should comment. I think provided one uses enough cortisone it does not really necessarily interfere with the reaction of the organisms to antibiotics.

*Dr. Gillespie:* The first specimen which we received was a swab from the wound where an intravenous cannula had been inserted before the patient arrived at the Bristol Royal Infirmary. At the time of the patient's arrival the wound was infected with a staphylococcus which we grew from the swab, and which evidently had been acquired at the other hospital. It was a fairly familiar type of staphylococcus, resistant to penicillin and tetracycline, phage pattern 80. Our next specimen was a blood culture taken when the patient had a rigor a day or two after operation, and this grew the same staphylococcus. The portal of entry in this case evidently was the infected vein. We also took a swab from the nose and found *Staph. aureus* in it; however, this had a different phage type and a different antibiotic sensitivity pattern and so had no connection with his blood stream infection.

*Mr. Peacock:* This patient had been on penicillin and streptomycin for a day or two before the operation.

*Dr. Gillespie:* Yes.

*Mr. Peacock:* Despite quite adequate doses of streptomycin to which the organism was sensitive it did not appear to prevent the spread of infection.

*Dr. Gillespie:* No, it is not easy to say why. Of course, the infection had started in the vein before the patient was given streptomycin. The dosage was not very great; it was an average sort of dose. And once an infection starts and becomes protected by thrombus and pus it may be difficult to destroy the organisms even with very energetic treatment by antibiotics. It must also be remembered that the disc-agar diffusion tests on which we rely for quick readings of antibiotic resistance in the laboratory are not very sensitive. I think it is possible to get a two-fold or even a four-fold difference in sensitivity between two strains without any clear difference in the disc test readings. For this reason we never like to rely on these tests alone when we are dealing with blood stream infections; we always confirm them by a quantitative agar dilution test. There was not time for a quantitative test on this occasion.

*Dr. Sanerkin:* Before I carried out the post-mortem examination I already knew that this patient had cirrhosis with portal hypertension, and that he also probably

PLATE XVIII

*Adult* Clonorchis sinensis. (× 10). *The coiled uterus is seen in the cephalic half (as a black coiled structure). In caudal part are the paired branched testes. Laterally are the vitelline ducts.*

PLATE XIX

*Operculated ovum of* Clonorchis sinensis (× 1500).

PLATE XX

*Intra-hepatic bile duct, showing mucous-gland hyperplasia in surrounding tissue* (× 75).

PLATE XXI

*Skin and subcutis from elbow, showing the unhealed venotomy incision and the thrombosed vein. Immediately deep to the incision there is an abscess, continuous with the thrombosed vein* (×7).

### PLATE XXII

*Heart opened to show the aortic valve. Vegetation affecting mainly the left coronary cusp; the tip of the vegetation is seen to pass into the left coronary ostium.*

### PLATE XXIII

*Transverse section across the main left coronary artery, blocked by vegetation. Irregular dark lines within the thrombus represent clumps of organisms* (× 9).

had clonorchiasis. He was a well-developed and well-nourished Chinaman. His skin was yellowish-brown, not entirely his natural colour because his conjunctivae were icteric. All over the body there were numerous small irregular cutaneous scars presumably as a result of the many eosinophilic abscesses he is known to have had. I think these eosinophilic abscesses have a common basis with the eosinophilia noted in peripheral blood and the eosinophilic infiltration in the liver.

He had an old splenectomy scar, and a healing thoraco-abdominal wound on the right side, in relation to which there was a right-sided fibrinous pleurisy. There were several venotomy incisions, some healed, some healing, and one in the right elbow which appeared not to have healed.

The post-mortem findings fall mainly into four groups: firstly the cirrhosis and portal hypertension, then the parasitic infestation of the biliary tract, thirdly the chronic gastric ulcer, and finally the staphylococcal infection.

The liver was cirrhotic with a finely nodular surface, and small regeneration nodules. The porto-caval anastomosis was quite patent and free from thrombus. The portal vein and its tributaries were thickened and dilated. The spleen had already been removed at a previous operation. There were well-developed anastomotic channels around the diaphragm, through the hiatus, and in the oesophageal submucosa. No bleeding point could be identified in the oesophageal mucosa. The stomach and intestines were full of blood.

Bearing in mind the possibility of parasitic infestation, I carefully "milked" the hepatic ducts before cutting into the liver and was able to recover several dozen flukes (Plate XVIII); these were about 1 cm long and 0·5 cm wide, and Dr. Crofton subsequently confirmed that they were indeed *Clonorchis sinensis*. Smears from the duct bile, which was very pale and mucoid, showed numbers of *Clonorchis sinensis* ova (Plate XIX). Sections from the larger intrahepatic bile ducts showed periductal mucous-gland proliferation which is a typical feature of clonorchiasis (Plate XX). The gall-bladder was greatly thickened and distended with mucoid bile, and contained an occasional fluke as well as ova. The pancreas showed squamous metaplasia with some dilation of its ducts. *Clonorchis* is known sometimes to invade the pancreatic ducts and squamous metaplasia is a common finding. In this case, however, no flukes were found in the pancreatic ducts.

A large deep active chronic peptic ulcer was found along the lesser curve of the stomach, with an eroded artery in its floor. This must have accounted for some of the alimentary haemorrhage, though not necessarily all, and he may well have been bleeding from the oesophageal varices as well.

A section from the unhealed venotomy wound showed staphylococcal thrombophlebitis (Plate XXI). Septic emboli from this vein lodged in a branch of the pulmonary artery, giving rise to a septic infarct of the lung. He also developed acute staphylococcal endocarditis of the aortic valve (Plate XXII). No pyaemic abscesses were found. Embolism from the aortic vegetations occurred to two sites: the first to the upper pole of the right kidney, producing a septic infarct; the second plugged the ostium and main part of the left coronary artery (Plate XXIII), causing sudden death from acute coronary occlusion.

The absence of generalized pyaemic abscesses is interesting, because I believe that in a staphylococcal endocarditis of several days' duration one might expect to find such abscesses, certainly in the kidney. The antibiotic treatment given probably proved efficacious in preventing fresh septic lesions of pyaemic type, but obviously failed to sterilize pre-existing lesions. I think this is because the bacteria were persisting and being harboured in inaccessible sites and non-viable tissue—in the thrombosed arm vein, in the infarcted lung and kidney, and finally within the thrombotic vegetation on the aortic valve.

*Mr. Peacock:* We had to make a decision as to whether the bleeding arose from the oesophageal varices or from the chronic gastric ulcer. It was not an easy decision to make but we did find that on releasing the oesophageal balloon and maintaining the pressure in the gastric balloon we could aspirate fresh blood from the oesophagus, whereas we could not aspirate any fresh blood from the gastric region. That led us to believe the blood in this particular patient was coming from the oesophageal varices.

The only other thing that has crossed my mind is the relationship of the clonorchis infection to the portal hypertension. I believe that this infection is present in at least half of the population of China and there is the same incidence in Japan.

*Dr. Coles:* May I ask Dr. Sanerkin if the valves where the bacterial endocarditis was were normal? Was it a normal aortic valve?

*Dr. Sanerkin:* There was no evidence of antecedent valve disease or abnormality; the valve was entirely normal.

*Professor Hewer:* Being an acute endocarditis it obviously is not necessary that the valve should have been previously abnormal.

*Dr. Gillespie:* Would Dr. Sanerkin like to say how long that endocarditis had been there?

*Dr. Sanerkin:* It is difficult to say. I think I must assume that the infection was more than 3–4 days old, though not much more. Certainly it had not produced any acute erosions of the valve cusp.

*Dr. Lloyd:* I would like to ask therefore whether a consideration of the time factor in this case might not help us in one or two respects. In the first place, was there really time for him to develop a pyaemia? And secondly was there really time for this staphylococcal infection to have been overcome by the antibiotics before the final catastrophe?

*Dr. Gillespie:* In answer to the second question, I think there probably was not time for the antibiotics to have overcome the staphylococcal infection to a degree that would have prevented the sudden obstruction of the mouth of the coronary artery. I am afraid I would not like to give an opinion as to how long it takes for pyaemia to develop. He had been running a low grade fever for approximately 1 month beforehand. From his temperature chart it appears that something fairly spectacular happened on the 16th.

*Dr. Lloyd:* Yes, that is only four days before he died, which corresponds exactly with Dr. Sanerkin's estimate of the age of the bacterial endocarditic lesion.

*Dr. Gillespie:* I would like to say that staphylococcal endocarditis which, as you all know, is a very lethal condition, requires the most energetic antibiotic treatment. It seems that the only hope is to hit the staphylococci very hard indeed as soon as one diagnoses or strongly suspects the condition. One would often recommend a double-barrelled or even a treble-barrelled attack for the first few days with two or three antibiotics in large doses. I have seen several cases of staphylococcal endocarditis, nearly all fatal, and have wondered afterwards whether we really gave them large enough doses of antibiotic. All the cases which I remember were given antibiotics in doses which would be considered adequate for ordinary staphylococcal infections. But these cases are much more difficult to treat. After all, even with subacute bacterial endocarditis due to a penicillin-sensitive *Streptococcus viridans*, very large quantities of penicillin have got to be given to ensure success. The patient was given penicillin and streptomycin, but since the organism was penicillin-resistant that in fact means streptomycin in a dose of 1 or 2 grams a day, which is not a very large dose.

*Professor Hewer:* These organisms had a thick layer of thrombus on top of them. You saw the vegetation in the coronary artery. I believe that the antibiotic diffuses slowly through the thrombus.

*Dr. Gillespie:* It looks as if the organisms must have been in the thrombus before treatment started and continued to grow there despite the antibiotics, first streptomycin alone and then some erythromycin as well.

*Professor Hewer:* I wonder if Dr. Crofton would like to tell us something about *Clonorchis sinensis*?

*Dr. Crofton: Clonorchis sinensis* has a high incidence in southern China and in Japan. There is a very nearly related form, *Opisthorchis tenuicollis*, much nearer home in East Prussia and other parts of Europe. Briefly, the life history is as follows: man becomes infected by eating infected fish. Eggs passed out in the faeces are eaten by a snail, larvae develop and escape from the snail into the water. These larvae invade a fish and pass into its musculature. If the infected fish is eaten by man, uncooked or only partially cooked (in parts of China this is considered a delicacy) then the larval stage develops into an adult. After the larva has been swallowed it passes mainly by the bile duct to the liver. One of the things about this case that interested me was the relatively small number of flukes which were present at post mortem. In fact there are numbers of something like 25,000 being reported from individuals with severe clonorchiasis and numbers something like 500 are considered to be a medium infection. Anything less, I gather, is more or less ignored in China.

*Peofessor Hewer:* We have not been told anything about the abscesses, how were they produced? Why should he have abscesses in the skin all over the body?

*Dr. Crofton:* I don't think this has anything to do with the clonorchiasis directly.

*Dr. Sanerkin:* An attempt was apparently made to explain this at the Hospital for Tropical Diseases in London; they decided it was not due to clonorchiasis, and thought it might be caused by nematode larvae of animal origin. I do not quite know what that might be; I did not find any intestinal worms.

*Dr. Crofton:* I have no evidence of any other infestation.

*Question:* Do you get any eosinophilia with this infestation?

*Dr. Sanerkin:* Eosinophilic reaction is not usual in clonorchiasis. Mr. Peacock pointed out that clonorchiasis is a widespread condition in China and Japan, and its association with cirrhosis is apparently an indirect one—the existence of two separate conditions. The main direct complications of clonorchiasis are said to be either infection of the bile ducts, i.e. acute cholangitis or cholangiolitis, or a bile duct carcinoma.

*Professor Hewer:* So we don't know why he had eosinophilia, which was a remarkable feature? It had nothing to do with the clonorchiasis.

*Dr. Crofton:* In heavier infections than this you do get biliary cirrhosis and involvement of adjacent liver parenchyma which undergoes pressure atrophy. In light infections such as this I would not expect cirrhosis.

*Professor Hewer:* Sheep get cirrhosis of the liver from ordinary flukes.

*Dr. Crofton:* Yes, but the position is different here. Sheep liver flukes have a very spiny cuticle. That of *Clonorchis* is smooth. The damage done by *Fasciola* is considerable even in light infections.

*Dr. Lloyd:* We hear that *Clonorchis sinensis* sometimes invades the pancreatic duct. Does it do that by accident or does it enjoy itself there?

*Professor Hewer:* Would anyone like to speak on the pastimes of a fluke?

*Dr. Crofton:* It has been reported that they invade the pancreas and pancreatic ducts, but such cases appear to be rare.

*Dr. Lloyd:* Does it ever cause pancreatic disease?

*Dr. Crofton:* I have no information about this in clonorchiasis.

*Question:* It is really true that this condition does not produce an eosinophilia?

*Dr. Crofton:* You do get eosinophilia in most parasitic infections. Eosinophilia in clonorchiasis does not appear to be a characteristic of the disease except in certain acute forms.

*Dr. Sanerkin:* Surely eosinophilia occurs in infestations in which the parasites or their products are found within the tissues of the body. Liver flukes live in the bile ducts, not in the tissues.

*Dr. Crofton:* Yes, although you do get some considerable reaction from the tissues in this case, particularly in the acute form.

*Professor Hewer:* There is no possibility at all that these eosinophilic abscesses could be anything at all to do with the clonorchiasis? No larvae that lost their way?

*Dr. Crofton:* I suppose it is just possible, but I do not think it likely.

*Professor Hewer:* Our present case does not help to settle this point about eosinophilia. There were eosinophilic abscesses but we are told they were not due to clonorchiasis. If something else produced the abscesses we cannot attribute the eosinophilia to clonorchiasis. It is a pity we have no evidence for the aetiology of these abscesses.

*Dr. Lloyd:* Is clonorchiasis normally a self-limiting disease? Can one get rid of these flukes or does the infestation persist indefinitely?

*Dr. Crofton:* The record is something like 25 years, but it is not quite certain whether the possibility of repeated infections has been eliminated in such estimates. It is self-limiting in the sense that very often people may lose their infections before they die.

*Dr. Peacock:* We also had another case of Dr. Read's, who was also employed in a Chinese restaurant.

*Dr. Crofton:* It is the snail host which is dangerous. There can be no direct cross-infection.

*Dr. Halford:* So we don't need to worry unless Chinese restaurants sell snails.

## Figures and Tables

**PLATE XVIII f1:**
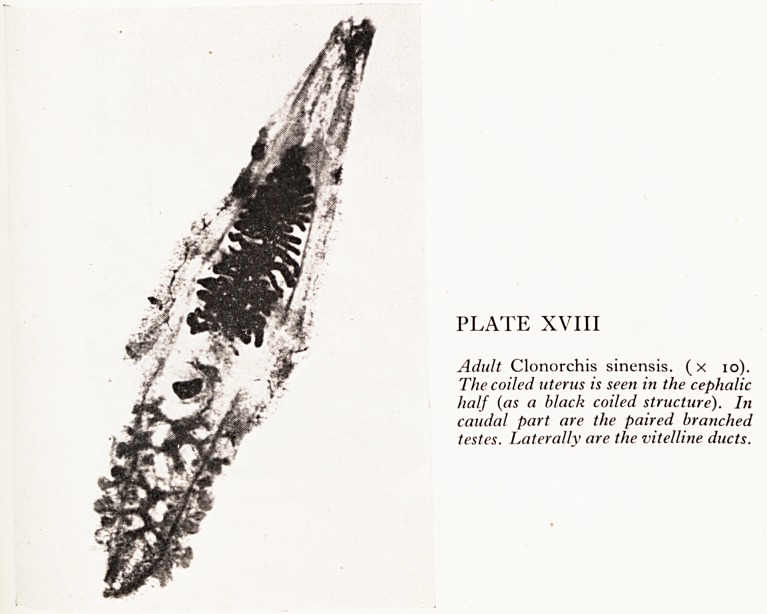


**PLATE XIX f2:**
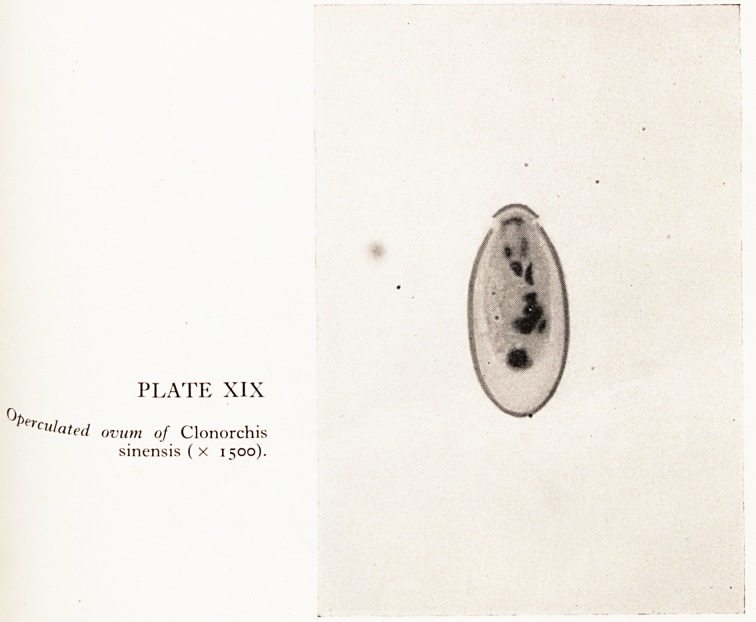


**Figure f3:**
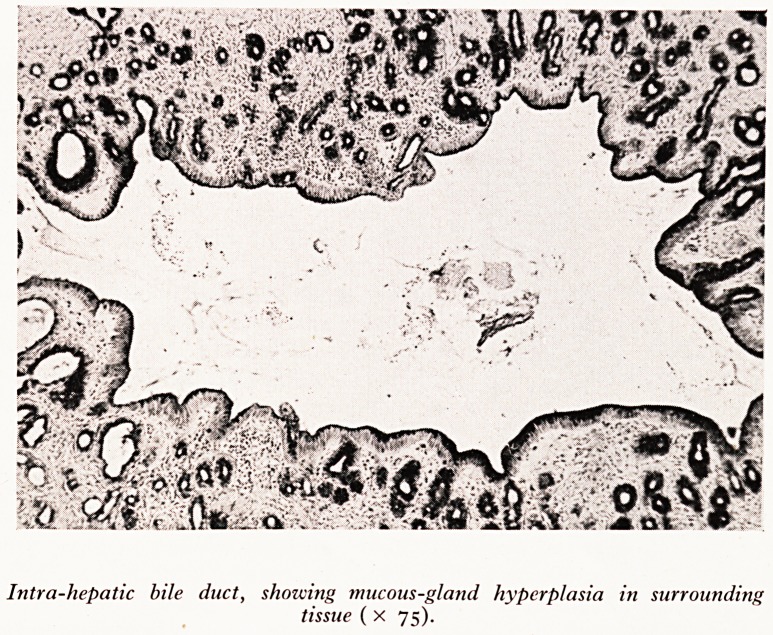


**Figure f4:**
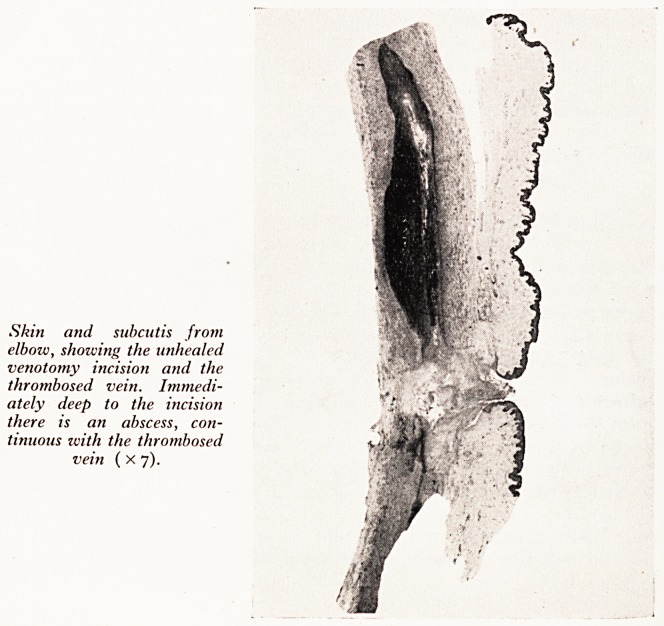


**Figure f5:**
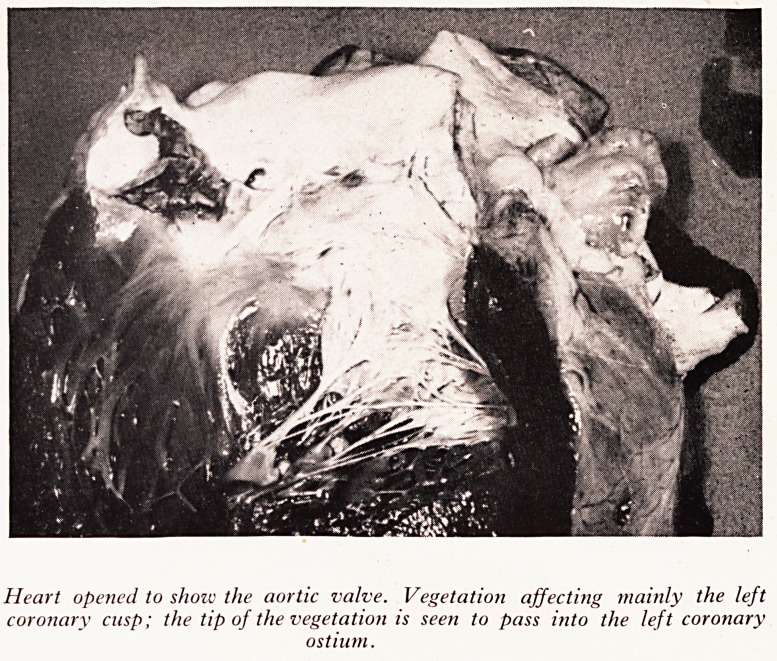


**Figure f6:**